# *In vitro* models to study viral-induced asthma exacerbation: a short review for a key issue

**DOI:** 10.3389/falgy.2025.1530122

**Published:** 2025-03-28

**Authors:** Rémi Pereira De Oliveira, Clément Droillard, Gilles Devouassoux, Manuel Rosa-Calatrava

**Affiliations:** ^1^ CIRI, Centre International de Recherche en Infectiologie, Team VirPath, Université de Lyon, Inserm, U1111, Université Claude Bernard Lyon 1, CNRS, UMR5308, ENS de Lyon, Lyon, France; ^2^ International Research Laboratory RESPIVIR France - Canada, Centre Hospitalier Universitaire de Québec- Université Laval, Québec, QC, Canada; ^3^ International Research Laboratory RESPIVIR France – Canada, Centre International de Recherche en Infectiologie, Université de Lyon, Inserm, U1111, Université Claude Bernard Lyon 1, CNRS, UMR5308, ENS de Lyon, Lyon, France; ^4^ Virnext, Faculté de Médecine RTH Laennec, Université Claude Bernard Lyon 1, Université de Lyon, Lyon, France; ^5^ Department of Respiratory Diseases, CIERA, Hôpital de la Croix-Rousse, Hospices Civils de Lyon, Université Claude Bernard Lyon 1, Lyon et CRISALIS/F-CRIN INSERM Network, Lyon, France; ^6^ Centre de Recherche en Infectiologie du Centre Hospitalier Universitaire de Québec - Université Laval, Faculté de Médecine, Département de Pédiatrie de l’Université Laval, Québec, QC, Canada

**Keywords:** asthma, viral infection, *in vitro* model, exacerbation, physiopathology

## Abstract

Asthma is a heterogenous inflammatory bronchial disease involving complex mechanisms, several inflammatory pathways, and multiples cell-type networks. Bronchial inflammation associated to asthma is consecutive to multiple aggressions on epithelium, such as microbiologic, pollutant, and antigenic agents, which are responsible for both T2 and non-T2 inflammatory responses and further airway remodeling. Because asthma physiopathology involves multiple crosstalk between several cell types from different origins (epithelial, mesenchymal, and immune cells) and numerous cellular effectors, no single and/or representative *in vitro* model is suitable to study the overall of this disease. In this short review, we present and discuss the advantages and limitations of different *in vitro* models to decipher different aspects of virus-related asthma physiopathology and exacerbation.

## 1 Introduction

Asthma affects more than 300 million individuals worldwide (1) and is characterized by airway obstruction, bronchial hyperresponsiveness, and bronchial inflammation. Asthma is schematically separated into two main immune and inflammatory-related phenotypes. The most prevalent is the T2-high phenotype, which involves allergic and/or eosinophilic inflammation, involving mainly Th2 lymphocytes, basophils, eosinophils, mast cells, and type 2 innate lymphoid cells (ILC2). By contrast, the T2-low inflammatory phenotype is driven by Th17 lymphocytes and/or neutrophils and is characterized by the absence of allergy, eosinophilic inflammation, and other T2 determinants, such as high FeNO levels, nasal polyposis, or atopic dermatitis (2). In some cases, when both neutrophil and eosinophil infiltration in the airways is low, the condition is called a mixed or pauci-granulocytic phenotype (3). A major burden of the disease is the occurrence of acute clinical events, known as asthma exacerbations, which are associated with an increase in bronchial inflammation in response to various environmental stimuli (4–6). Among these stimuli, allergens, pollutants, bacteria, and viruses are largely implicated and responsible for initiating the inflammatory cascade. They activate three main epithelial alarmins: thymic stromal lymphopoietin (TSLP), IL-33, and IL-25, which activate dendritic cells, ILC2, macrophages, T cells, and mast cells (7, 8). Respiratory viral infections are a leading cause of asthma exacerbation, with human rhinovirus (HRV) being the most commonly implicated, followed by respiratory syncytial virus (RSV) (9) and, to a lesser extent, human metapneumovirus (hMPV) and influenza viruses (9, 10). The virus-related mechanisms of asthma exacerbation are not yet fully understood and the development of relevant *in vitro* models to study virus/asthma functional relationships remains a significant challenge. In this short review, we summarize and discuss the advantages and limitations of the commonly used *in vitro* models for studying asthma and its exacerbation by viral infections.

## 2 Monoculture cell line models

The simplest models involve human bronchial cell lines cultured in medium or at the air–liquid interface (ALI). Some of these cell lines, such as CaLu-3 and 16HBE, express tight junction (TJ) proteins, which are key determinants of epithelium permeability and integrity (11, 12). Disrupted TJs are observed in the bronchial epithelium of asthmatic patients, significantly affecting respiratory airway barrier function (13). Studies using allergens, nanoparticles, or cigarette smoke extract have shown an increase in transepithelial permeability through the disruption of TJs on CaLu-3 (14, 15) and 16HBE (16, 17). Therefore, measuring TJ integrity and exploring functional interactions with respiratory viruses in permissive epithelial cells (Table 1) should be considered, especially as HRV, RSV, hMPV, influenza, and coronaviruses are known to target specific TJ proteins (18–21). However, although airway epithelial cell lines are more accessible than primary cells and can be cultured in ALI models to study host responses to viral infections (22), they are less physiologically relevant due to the lack of cell diversity (i.e., only ciliated cells are present, with no secretory or basal cells) (23, 24) and the absence of cell lines from asthmatic patients.

**Table 1:** Common cells used in asthma studies and susceptible to respiratory viruses.

Cell lines	Can be infected by	References
CaLu-3	RSV, HRV, SARS-CoV, Influenza	(22, 25–27)
16HBE	RSV, HRV, SARS-CoV-2, Influenza, HMPV	(20, 28–31)
PBEC	RSV, HRV, SARS-CoV-2, Influenza, HMPV	(32–36)

## 3 Primary cell-based ALI human airway models

Primary bronchial epithelial cells (PBECs) are more relevant models, as they are directly recovered from patient biopsies, including those with asthma or other pulmonary diseases such as chronic obstructive pulmonary disease (COPD). PBECs can be cultured in submerged mono-cultures or in an air–liquid interface as a pseudostratified human airway epithelium (HAE) that includes several types of differentiated cells (ciliated, club, goblet, and basal) (37). These models allow for the investigation of a broader range of functional and physiological characteristics, including morphology, transepithelial electrical resistance, tight junction integrity, mucus secretion, cilia clearance, innate immune response (38, 39), sensitivity to allergens or respiratory viruses (Table 1), and transcriptomic signatures (40). As such, PBECs are widely used to study asthma physiopathology and exacerbation (41, 42). For example, the T2 associated IL-13 cytokine has been extensively studied for its ability to modulate gene expression profiles (43) and alter mucin production in primary cell-based ALI models (44), which are features of asthma physiopathology.

Moreover, HAE models are well-suited for investigating asthma exacerbation related to viral infection, as asthmatic patients exhibit physiological impairments that can be measured in HAE. These impairments include alterations in tight junctions and epithelium integrity (20, 21, 45), modifications in mucus production, mucociliary function (46–49), and aberrant epithelial tissue repair and remodeling through epithelial-mesenchymal transition (50–52).

Furthermore, PBECs can also react to viral infections through the innate immune response, which is an important factor in asthma exacerbation (8, 53, 54), as respiratory viruses are known to suppress antiviral responses and enhance inflammation in epithelial cells (55–57). For example, Wark et al. demonstrated an impairment of interferon beta (IFN-β) in ALI cultures from asthmatic patient PBECs compared to healthy controls during HRV infection (36). Consistently, the addition of IFN-β restored the expression of antiviral genes against HRV in asthmatic PBECs, thereby reducing the duration of inflammation (58). In addition, viral double-stranded RNA induces a stronger expression of the alarmin TSLP, an important enhancer of asthma exacerbation through the induction of the T2 inflammatory pathway, in asthmatic PBEC-based ALI compared to healthy controls (59). These results are similar to those obtained with HAE models infected by different respiratory viral infections, such as RSV (60), HRV (61), and SARS-CoV-2 (62).

## 4 ALI models co-cultivated with primary cells of different origins

Asthma is a complex lung disease that involves not only epithelial cells but also immune cells implicated in inflammation, viral clearance, and remodeling processes. In addition, mesenchymal cells, such as fibroblasts, endothelial cells, and smooth muscle cells, contribute to tissue inflammation and physiopathology (63). In this context, co-cultured primary cells of different origins can be very useful for investigating cellular crosstalk and their respective contributions to asthma development and exacerbation (Table 2). The ability of respiratory viruses to impair host immune responses, thereby increasing the risk of asthma exacerbation (64–67), underscores the need for further investigation into the functional relationships between epithelial, mesenchymal, and immune cells within integrated *in vitro* asthma models. In this regard, Transwell® cell separation system used for ALI cultures (68) allows for the co-cultivation of epithelial cells in the open air with other types of primary cells, whether adherent or not, on the lower surface of the Transwell®. Furthermore, co-culture models with direct contact between primary cells can be highly beneficial for mimicking *in vivo* crosstalk.

**Table 2:** 3D co-culture models used to study asthma physiopathology and exacerbation.

3D co-cultures models	Cell origins	Conclusions	References
PBEC and fibroblast	Healthy donor cells	Activation of smooth muscle through TGF-*β*2 secreted from injured epithelial cells	(69)
BEAS-2B or PBEC and airway smooth muscle cells	Healthy donor for smooth muscle cells	Proliferation of smooth muscle and stimulation of pro-inflammatory genes expression	(70)
16HBE and HUVECs cells	Cell lines	Proliferation of endothelial cells and expression of E-selectin and ICAM-I	(71)
PBEC and macrophages	Healthy donor cells	Inflammation and viral clearance	(72)
PBEC and macrophages and dendritic cells	PBECs from healthy, asthmatic, COPD donors	Inflammation	(73)
PBEC and fibroblast and eosinophils	Healthy donor cells	Epithelium remodeling and inflammation	(74)
PBEC and PBMC/lymphocytes	Healthy donor cells	Inflammation, exacerbation and viral clearance	(75–77)
PBEC and neutrophils	Healthy donor cells	Neutrophils infiltration induced by HRV infection	(78)
Organoids and macrophages	Healthy donor cells	Increase of pro-inflammatory proteins with macrophages	(79)
Organoids and resident T lymphocytes	Healthy donor cells	T lymphocytes activation after SARS-CoV-2 infection	(80)

PBEC, primary bronchial epithelial cell; PBMC, peripheral blood mononuclear cell.

### 4.1 Co-culture of ALI airway models with mesenchymal cells

ALI co-culture models of healthy airway epithelium with fibroblast cells adhering to the lower surface of Transwell® have highlighted that chemical injury to the epithelium leads to the secretion of TGF-β2 by fibroblasts, which in turn triggers the expression of several asthma markers, such as Tenascin-C and α-smooth muscle actin (α-SMA) (69), as observed in the bronchus of asthmatic patients (81). In a similar model, Reeves et al. reported that fibroblasts secrete more hyaluronan when co-cultured with asthmatic ALI airway epithelium compared to healthy tissue, thereby promoting inflammation by increasing leukocyte binding. In this study, bronchial epithelial cells were co-cultured with human lung fibroblasts with Fibroblast Growth Media (FGM-2) and PneumaCult ALI for 96 h (82). Consistently, fibroblasts infected by RSV also secreted more hyaluronan, promoting mast cell binding and protease production, which are both known to contribute to asthma inflammation (83).

Co-culture models of epithelial cells with healthy smooth muscle cells in various culture media have been developed to study cell proliferation and pro-inflammatory responses (70), in line with their role in asthma physiopathology through bronchoconstriction and airway tissue remodeling (84). In addition, the monocyte migration chemokine CCL5 was reported to be significantly more expressed in asthmatic smooth muscle cells than in healthy ones when the ALI model was infected by HRV. This aligns with the increased risk of asthma exacerbation observed through monocyte migration in infected asthma patients (85). On the other hand, co-culturing asthmatic smooth muscle cells with bronchial epithelial cells increases their susceptibility to HRV infection compared to co-cultures with healthy smooth muscle cells, with an increased expression of CCL20 by smooth muscle cells altering the antiviral PKR/eIF2 pathway within epithelial cells (86).

Endothelial cells are also important for tissue inflammation by binding factors on the surface membrane that enhance leukocyte infiltration (87). A co-culture model involving epithelial 16HBE and endothelial HUVECs, stimulated with virus mimicking double-stranded RNA, showed an increased expression of leukocyte-recruiting adhesion molecules (E-selectin and ICAM-I) on endothelial cells. In this study, Blume et al. co-cultured human bronchial epithelial cells in minimum essential medium (MEM) with Glutamax and serum, and HUVEC cells in M199 medium supplemented with L-Glutamine seeded at the basal side, for up to 8 days (71). Similar results were obtained when healthy ALI cultures were infected with SARS-CoV-2 (88).

Finally, ALI co-culture models are not only suitable for studying crosstalk between epithelial and mesenchymal cells involved in bronchial inflammation and immune cell recruitment, but they are also essential for characterizing the epithelium’s sensibility to viral infections. This makes them highly interesting for deciphering virus-related mechanisms that lead to asthma exacerbation.

### 4.2 Co-culture of ALI airway models and immune cells

ALI airway models co-cultured with additional immune cell components are even more suitable for studying the mechanisms underlying asthma physiopathology. After initial epithelial cell injuries, immunes cells are recruited, activated, and amplify bronchial inflammatory cascades that can lead to asthma exacerbation and tissue remodeling. In this context, M1- or M2-type polarized macrophages contribute to determining the asthma phenotype (89) and exacerbation in cases where viral clearance is delayed (90).

Using Transwell® co-culture, Ronaghan et al. co-cultured human bronchial epithelial cells in ALI with macrophages in PneumaCult ALI medium or ImmunoCult-SF Macrophage Medium (IMM) for 72 h (72). They reported that M1 macrophage polarization reduced RSV infection in healthy PBECs ALI cultures, but not M2 polarization (72). These results are consistent with the predominance of M2 macrophages in asthmatic patients (91), which are associated with decreased *in vivo* interferon secretion and the promotion of T2 cytokines. This could help explain why asthmatics are more susceptible to RSV infection and related asthma exacerbation (92).

Other studies have developed more complex models by co-cultivating three different cell types. Paplinska-Goryca et al. used PBECs from healthy, asthmatic, or COPD patients in co-culture with macrophages on apical side and dendritic cells in the lower surface of Transwell®. This model highlighted the importance of immune cells and the origin of PBECs in the expression of alarmins when epithelium was exposed to double-stranded RNA stimulation (93). Similarly, Choe et al. showed the importance of eosinophils in airway epithelium remodeling and inflammation through a co-culture model of epithelial and fibroblast cells with eosinophils. Briefly, eosinophils, isolated from human venous blood, were seeded on top of the epithelium with RPMI 1640 medium. After 2 h of incubation and medium removal, eosinophils were stimulated with calcium ionophore for 48 h of co-culture (74). This co-culture model provides information about lung epithelium remodeling mechanisms in asthmatics (51).

Co-culturing peripheral blood mononuclear cells (PBMCs) at the lower surface of the Transwell® allows for the inclusion of a broader range of immune cells, enabling the study of crosstalk between epithelial cells and lymphocytes or natural killer (NK) cells. Several studies have focused on the T1/T2 balance and inflammatory cytokines, such as IP-10 and IFN-*γ*, during influenza and HRV infections of healthy nasal epithelium co-cultivated with PBMC in RPMI supplemented with serum for 48 h (75, 76). Qin et al. reported that RSV infection of healthy bronchial ALI cultures promotes an increase in Th2 cell populations from co-cultivated PBMC (77). Although NK cell polarization is known to be important for viral clearance and inflammation control through the secretion of IFN-*γ* or IL-4 and by killing eosinophils and neutrophils (94–96), their role in asthma physiopathology and exacerbation is not fully understood. To explore this further, co-culture models of human nasal epithelial cells with PBMC have been used to highlight NK cell activation after influenza infection (76). Future co-culture models with HAE and purified NK cells are needed to further study their role in asthma exacerbation during viral infections.

## 5 Organoids

Organoids are three-dimensional structures derived from stem cells or induced pluripotent stem cells, which harbor some of the structural and functional characteristics of organs, including the bronchus, to study airway development and physiopathology (97–100). These organoids are spherical, with a lumen at the sphere’s center or epithelial cells exposed at the surface (101), allowing them to be permissive to respiratory viruses like RSV (102, 103), SARS-CoV-2 (102, 104, 105), HMPV (106), influenza (104), or human parainfluenza 3 virus (107). Studies using healthy organoids have focused on aspects of asthma pathology, including goblet cell metaplasia (108) and remodeling (109).

Organoids co-cultured with immune cells are also being developed to mimic functional cell–cell interactions. Various methods have been explored: (i) seeding immune cells and lung stem cells together, (ii) co-cultivation of organoids with isolated immune cells, (iii) injecting immune cells into the organoid lumens, and (iv) expanding cultures from tissue samples that include both epithelial and resident immunes cells (110). Seo et al. co-cultivated macrophages with organoids and stimulated them with lipopolysaccharide (LPS) to model alveolar inflammation. They showed an increase in pro-inflammatory protein expression in the presence of macrophages, supporting the relevance of such organoid models (79). Organoids with resident immune cells have also been used to study SARS-CoV-2 infection and the corresponding T lymphocyte response (80), making these models promising for studying the crosstalk between resident immune cells and lung tissue. However, the development of organoid models using cells from asthmatic patients is currently lacking whereas they would be useful to complement the ALI models to further decipher viral mediated mechanisms associated with this pathology.

## 6 Lung-on-chip

Microfluidic systems, known as lung-on-chip models, are more complex systems that enable the movement of cells and media on the basal side, or the exposure of stimuli by injecting air onto the apical side of the airway (111, 112). For example, a lung-on-chip system with a co-culture of lung epithelium and circulating neutrophils in the basal medium was used to explore the mechanism behind asthma exacerbation induced by HRV infection. Briefly, the epithelium was cultivated in ALI on the surface of the chip membrane. On the other side, endothelial cells were cultivated in the vascular channel of the chip. After HRV inoculation at the apical side of the epithelium, neutrophils isolated from human peripheral blood were added to the chip’s reservoirs and perfused in RPMI 1640 (78). Studies have shown that neutrophil infiltration into the epithelium, a factor of neutrophilic asthma exacerbation, was induced by HRV infection and significantly increased with IL-13 stimulation (78). Another innovative system, known as “breathing lung-on-cheap,” mimics breathing and has been used to evaluate the effects of toxic particles potentially involved in the development and/or exacerbation of asthma (113). This model could be interesting when studying the impact of respiratory volume on asthmatic epithelium infection and asthma exacerbation. However, further development is needed to incorporate cells from patients.

## 7 Discussion

**Figure 1:**
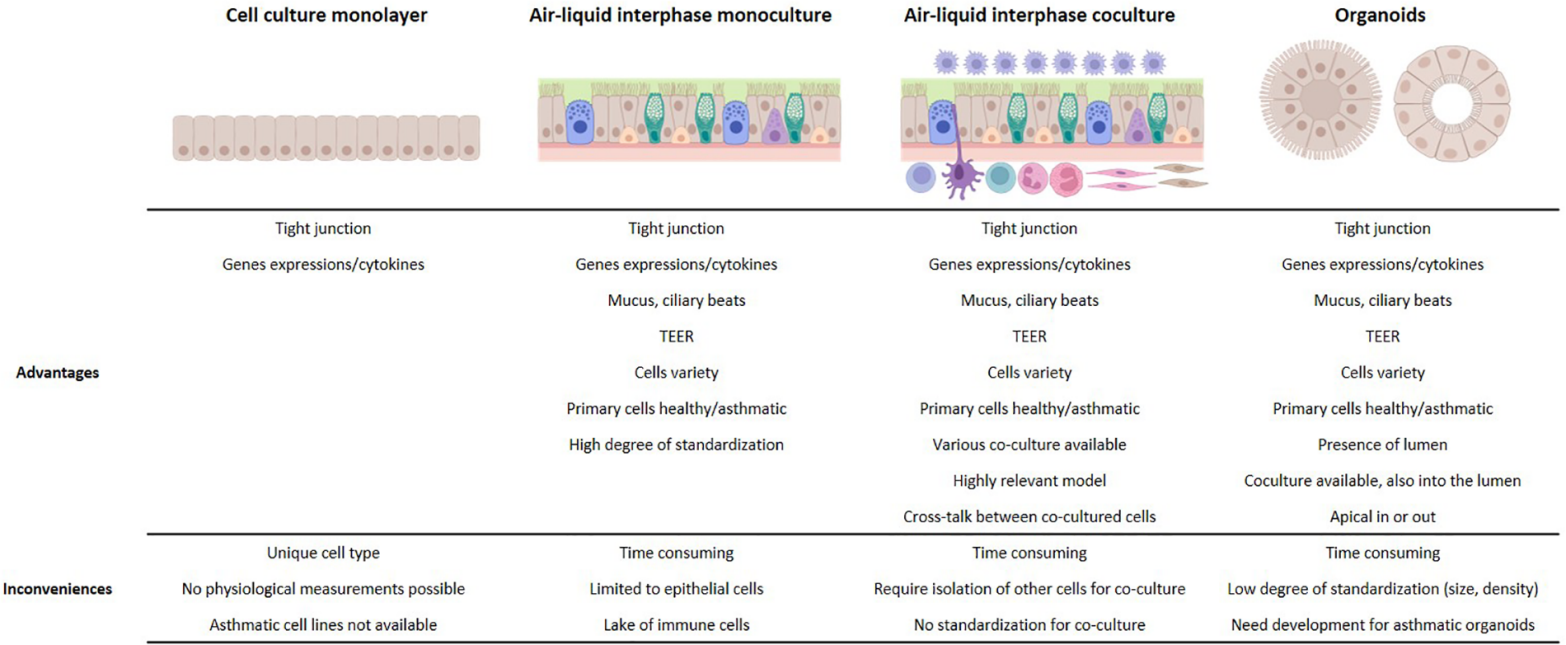
Advantages and limitations of *in vitro* models for the study of asthma exacerbation.

*In vitro* 3D airway models offer significant advantages compared to monolayer cell line-based *in vitro* models (Figure 1). Airway epithelial functions, including relevant inflammatory and antiviral responses, are reconstituted with a large variety of primary cells from healthy individuals of different ages and/or from patients with various bronchial diseases, such as asthma or COPD. This makes ALI and organoid models valuable and complementary tools for studying the complex mechanisms involved in asthma and its exacerbations. However, further development of and characterization of co-culture models involving mesenchymal and immune cells are needed to better understand key aspects of asthma physiopathology, such as inflammation, immune cell polarization, and tissue remodeling, leading to the development of new therapeutic approaches.

In conclusion, ALI co-culture models, organoids, and lung-on-chip systems are highly effective tools for studying asthma and its exacerbation from viral infections. With a diverse range of models available, each offering unique strengths and limitations, the choice of the most suitable *in vitro* model will depend on its relevance and ability to complement others in revealing various aspects of virus-induced asthma pathophysiology and exacerbation.
